# Mitochondrial DNA copy number as a mediator of the relationship between insulin resistance and cognitive function in patients with euthymic bipolar disorder

**DOI:** 10.1002/kjm2.12914

**Published:** 2024-11-23

**Authors:** Tsung Han Tsai, Cheng Ying Wu, Cheng Chen Chang, Ta Tsung Lin, Chin San Liu, Po See Chen

**Affiliations:** ^1^ Department of Psychiatry, National Cheng Kung University Hospital, College of Medicine National Cheng Kung University Tainan Taiwan; ^2^ School of Medicine Chung Shan Medical University Taichung Taiwan; ^3^ Department of Psychiatry Chung Shan Medical University Hospital Taichung Taiwan; ^4^ Vascular and Genomic Center, Institute of ATP Changhua Christian Hospital Changhua Taiwan; ^5^ Department of Neurology Changhua Christian Hospital Changhua Taiwan; ^6^ Graduate Institute of Integrated Medicine China Medical University Taichung Taiwan; ^7^ Department of Post‐Baccalaureate Medicine, College of Medicine National Chung Hsing University Taichung Taiwan; ^8^ Institute of Behavioral Medicine, College of Medicine National Cheng Kung University Tainan Taiwan

**Keywords:** bipolar disorder, cognition, executive function, insulin resistance, mitochondrial DNA copy number

## Abstract

Persistent cognitive challenges in bipolar disorder (BD) may be tied to insulin resistance, which crucially affects both metabolism and brain health. Additionally, mitochondrial DNA (mtDNA) copy number has emerged as an indicator of cognitive performance and response to treatment in BD. However, it remains unclear whether and how this indicator might serve as a bridge between metabolic dysfunction and cognitive capacity. In 68 study participants with euthymic BD, insulin resistance was assessed according to fasting glucose and insulin levels. mtDNA copy number was quantified from leukocytes, and executive function was measured with the Wisconsin card‐sorting test (WCST). Mediation models were applied to explore the statistical relationship between insulin resistance, mtDNA copy number, and executive function. Both linear regression and Poisson distribution approaches with robust bootstrap simulations were used for significance testing. The results indicated that insulin resistance indirectly affects executive function via mtDNA copy number. This mediation relationship was statistically significant for both preservation errors and completion of categories in the WCST, although there were no significant direct effects of insulin resistance on the executive functions. We therefore concluded that insulin resistance is associated with reduced mtDNA copy number in blood, which may negatively impact executive functions in patients with euthymic BD. Further work is warranted to determine if improving metabolic and mitochondrial health may lead to better cognitive outcomes in BD.

## 1 INTRODUCTION

Patients with bipolar disorder (BD) often experience cognitive deficits that persist even during periods of mood stability. The link between insulin resistance and cognitive impairment in BD patients is an area of growing interest, partially because metabolic syndrome is highly prevalent in BD patients^1^ and also because insulin is a critical regulator of both metabolism and brain function. Sasaoka et al. explored likely mechanisms underlying the relationship between insulin resistance and cognition, identifying amyloid‐beta oligomers, tumor necrosis factor‐alpha (TNF‐alpha), JNK activation, and orexin function as key factors.^2^ Moreover, our previous work highlighted the potential negative impact of insulin resistance on the cognitive performance of BD patients.^3^, ^4^ Collectively, these studies underscore the importance of managing insulin resistance to preserve cognitive function.

Mitochondrial DNA (mtDNA) is prone to oxidative damage due to its location in the mitochondrial matrix, where reactive oxygen species are often generated in abundance. Furthermore, the mtDNA copy number (mtDNA CN) changes in response to oxidative stress and inflammation, conditions that are implicated in mitochondrial dysfunction. Variations in mtDNA CN have been linked to several psychiatric disorders, including BD, which suggests mitochondrial dysfunction may play some role in these conditions. Previous studies have highlighted the role of mitochondrial dysfunction in cognitive impairment, particularly in the context of major depressive disorder.^5^ Similarly, our previous studies examined how mtDNA CN correlates with cognitive ability in euthymic BD patients undergoing treatment with valproate.^6^ We identified a pronounced elevation in mtDNA CN in BD patients compared to controls, and this disparity remained even when controlling for factors such as age, sex, and metabolic syndrome. Additionally, we found an apparent positive relationship between valproate blood levels and mtDNA CN in the BD patients. When we further investigated how mtDNA CN levels might influence the clinical characteristics of BD, we found that mtDNA CN could be used as a potential biomarker to distinguish between individuals who did and did not respond to valproate treatment. In particular, those with higher mtDNA CN levels were more likely to have responded to valproate and exhibited improved cognitive function. The insights gained from this study underscored the potential of mtDNA CN as a biomarker for both treatment responsiveness and cognitive assessments in BD.

In both psychiatric and non‐psychiatric populations, insulin resistance has been associated with indicators of mitochondrial dysfunction, including changes in mtDNA CN. Evidence suggests that insulin resistance may lead to mitochondrial stress and impair mitochondrial biogenesis, resulting in altered mtDNA CN.^7^ Furthermore, insulin resistance is associated with high oxidative stress, and studies have shown that oxidative stress‐mediated damage to mtDNA can lead to reduced mitochondrial function and lower mtDNA CN.^8^ Insulin resistance‐associated glucotoxicity and lipotoxicity may also compromise mitochondrial health, contributing to the pathophysiology of cognitive impairment.^9^ This connection is evident in psychiatric populations, where mitochondrial dysfunction and altered mtDNA CN are commonly observed in conditions such as BD.^10^ Taken together, the existing literature suggests that the relationship between insulin resistance and mitochondrial dysfunction is complex, potentially bidirectional, and may involve disruptions in cellular energy homeostasis that impact mtDNA stability.^11^

The objective of this work was to ascertain whether mtDNA CN can serve as an intermediary factor linking insulin resistance to cognitive ability in patients with euthymic BD. The underlying hypothesis is that metabolic imbalances affecting insulin sensitivity may influence cognitive function, and mtDNA CN may act as an important mediator of this dynamic.

## 2 MATERIALS AND METHODS

### 2.1 Participants

Research on human subjects was conducted at National Cheng Kung University Hospital and received approval from the Institutional Review Board (IRB no. A‐ER‐104‐031). Participants were recruited from the outpatient department, and each provided written consent to participate. Sixty‐eight participants with euthymic BD were recruited. The study population included adults ranging from 18 to 70 years old. Diagnosis of BD was based on an interview with a psychiatrist using the Modified Schedule for Affective Disorders and Schizophrenia–Lifetime (Chinese Version). This tool aligns with Diagnostic and Statistical Manual of Mental Disorders, Fifth Edition (DSM‐5) criteria and is noted for its reliability of interrater agreement. Patient mood was assessed with the Hamilton Depression Rating Scale (17 items) and the Young Mania Rating Scale (11 items). Exclusion criteria were implemented by examining medical records and self‐reported surveys to eliminate variables affecting mitochondrial function. These criteria ruled out individuals with significant surgical or physical conditions, heart disease, stroke, renal conditions requiring dialysis or transplantation, cerebrovascular, neurodegenerative, or macrovascular disorders.

### 2.2 Insulin resistance

Fasting plasma glucose (FPG) and fasting serum insulin (FSI) levels of all participants were measured in one laboratory using the same assays to ensure consistency. Insulin resistance was estimated using the homeostatic model assessment of insulin resistance (HOMA‐IR) equation [HOMA‐IR = FPG (mmol/L) × FSI (U/mL)/22.5].

### 2.3 Mitochondrial DNA copy number

A FlexiGene DNA Kit (Qiagen, Hilden, Germany) was used to extract genomic DNA from the buffy coat, and all procedures followed the instructions of the FlexiGene DNA Handbook. The methods for testing leukocyte mtDNA CN and oxidative damage were performed as described previously.^12^ Leukocyte mtDNA CN was assessed using a LightCycler instrument (Roche, Mannheim, Germany). Briefly, polymerase chain reaction (PCR) was performed on sample DNA to amplify the ND1 gene in mtDNA and the β‐globin gene in nuclear DNA. The mtDNA CN was calculated by interpolation from the linear region of a standard curve made using plasmid containing the ND1/β‐globin genes.

### 2.4 Wisconsin card‐sorting test

The Wisconsin card‐sorting test (WCST) was used to assess executive function. Participants completed a computerized version of the WCST with 64 cards under the supervision of an experienced clinical neuropsychologist. Participants were required to sort cards according to one of three dimensions (color, shape, or number) on the basis of sign feedback without being given information about the dimensions. After sorting a series of 10 cards in one category, the subject was asked to sort the cards again in a different category. The indexes of preservative errors and completed categories were used for subsequent analyses.

### 2.5 Statistical analysis

**FIGURE 1:**
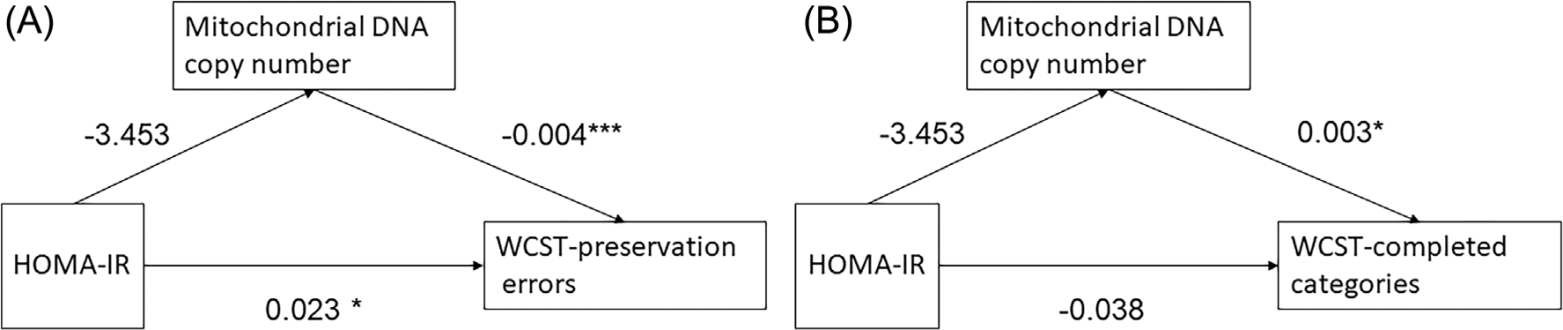
Visualization of mediation models with coefficient estimates of mediator and outcome models. *p < 0.05, ***p < 0.001. HOMA‐IR, homeostasis model assessment of insulin resistance; WCST, Wisconsin card‐sorting test.

The mediation analyses were performed using R software (open source and version 4.2.2), while other analyses were conducted using SPSS Statistics 20.0 (SPSS Inc.). Using a model‐based approach implemented in R package mediation v.4.5.0,^13^ causal mediation analyses were performed with the following models: (a) mediator model: mtDNA CN  ~ HOMA‐IR; (b) outcome model: indexes of WCST ~ mtDNA CN + HOMA‐IR, with HOMA‐IR as the independent variable and mtDNA CN as the mediator (Figure 1). The mediator model was fit with a linear regression model, and the outcome model was fit with a Poisson distribution because the indexes of WCST were considered to be count variables. A nonparametric bootstrap procedure with 10,000 simulations was used to calculate significance. Sensitivity analysis for sequential ignorability was performed to assess the potential effects of unmeasured confounders.

## 3 RESULTS

Table 1 shows the descriptive characteristics of the study population as well as the mtDNA CN measurements (mean, standard deviation (SD)) and WCST indexes. Figure 1 presents the coefficient estimates of the mediator model and outcome model. There are two parts to the WCST results: preservation errors and completed categories. mtDNA CN was inversely correlated with WCST preservation errors (*r*[67] = −0.0038, *p* < 0.001) and positively correlated with WCST completed categories (*r*[67] = 0.0025, *p* = 0.028). HOMA‐IR was positively correlated with WCST preservation errors (*r*[67] = 0.023, *p* = 0.013) but not significantly associated with mtDNA CN or WCST completed categories.

**TABLE 1:** Demographics, participants.

Variable	Total sample (*n* = 68)
	*N* (%)
Sex, female	27 (40%)
	Mean (SD)
Age	42.38 (13.82)
Education (years)	14.42 (2.57)
BMI (kg/m^2^)	26.45 (4.95)
Young mania rating scale	0.35 (0.94)
Hamilton depression rating scale	1.75 (2.06)
HOMA‐IR	3.93 (3.38)
mtDNA CN	100.78 (55.90)
WCST_preservative errors	13.49 (10.89)
WCST_completed categories	2.46 (1.71)

Abbreviations: BMI, body mass index; HOMA‐IR, homeostasis model assessment of insulin resistance; mtDNA CN, mitochondrial DNA copy number; WCST, Wisconsin card sorting test.

Table 2 shows the analysis results of mediation models exploring the relationship between insulin resistance (measured by HOMA‐IR), mtDNA CN, and executive functions assessed by WCST. For WCST preservation errors, the indirect effect of HOMA‐IR on executive functions through mtDNA CN (average causal mediation effect [ACME]) was statistically significant, with an estimated effect of 0.15 and a 95% bias‐corrected bootstrap confidence interval (BCa CI) from 0.02 to 0.40 (*p* = 0.017). The direct effect of HOMA‐IR on executive functions without mtDNA CN (average direct effect [ADE]) was not statistically significant, with an estimated effect of 0.26 and a 95% BCa CI from −0.35 to 0.75 (*p* = 0.32). The total effect, which combines ACME and ADE, was not statistically significant, with an estimated effect of 0.42 and a 95% BCa CI from −0.15 to 0.92 (*p* = 0.13). The proportion of the total effect that is mediated by mtDNA CN (proportion mediated) was 0.37.

**TABLE 2:** Results of the mediation models involving, homeostasis model assessment of insulin resistance, mitochondrial DNA copy number and indexes of Wisconsin card‐sorting test (WCST).

	Mediation model					
	WCST—preservation errors			WCST—completed categories		
	Estimated effect	95% BCa CI	*p*	Estimated effect	95% BCa CI	*p*
ACME	0.15	0.02, 0.40	0.017*	−0.03	−0.06, 0.00	0.037*
ADE	0.26	−0.35, 0.75	0.32	−0.11	−0.52, 0.06	0.24
Total effect	0.42	−0.15, 0.92	0.13	−0.14	−0.56, 0.03	0.14
Proportion mediated	0.37			0.19		

Abbreviations: ACME, average causal mediation effects; ADE, average direct effects; BCa CI, bias‐corrected bootstrap confidence intervals.

* *p* < 0.05.

For WCST completed categories, the ACME was statistically significant, with an estimated effect of −0.03 and a 95% BCa CI from −0.06 to 0.00 (*p* = 0.037). The ADE was not statistically significant, with an estimated effect of −0.11 and a 95% BCa CI from −0.52 to 0.06 (*p* = 0.24). The total effect was not statistically significant, with an estimated effect of −0.14 and a 95% BCa CI from −0.56 to 0.03 (*p* = 0.14). The proportion mediated was 0.19.

Overall, these results suggest that mtDNA CN may play a role in mediating the impact of insulin resistance on executive functions in euthymic BD patients, particularly functions related to preservation errors in the WCST.

## 4 DISCUSSION

In this study on euthymic BD patients, we assessed whether mtDNA CN may act as a mediator of the relationship between insulin resistance and cognitive function. Our findings suggest that insulin resistance detrimentally impacts executive function in part by reducing mtDNA CN. Hence, insulin resistance and mtDNA CN may serve as non‐invasive, modifiable targets for interventions aimed at halting neuroprogression in BD patients.

Previously, we established a link between insulin resistance and compromised executive function in euthymic BD patients.^4^ Intriguingly, mitochondrial dysfunction (as indicated by mtDNA CN variations) has been associated with both insulin resistance and cognitive impairment in the general population.^7^, ^14^ Recent literature also suggests that mtDNA CN alterations occur in BD and neurocognitive disorders, and these associations are related to disease pathology.^15^, ^16^, ^17^ Based on these studies, we postulated that mitochondrial dysfunction might mediate the relationship between insulin resistance and cognitive function in BD. In this context, mtDNA CN alterations may contribute to cellular mechanisms such as impaired energy production, increased oxidative stress, and activation of cell death pathways.

The association between insulin resistance and mitochondrial dysfunction is complex and possibly bidirectional.^7^ Moreover, environmental factors may impact mitochondrial function via different mechanisms associated with glucotoxicity and lipotoxicity.^10^ While previous clinical studies showed that insulin resistance is correlated with low mtDNA CN, a similar association was not observed in our current dataset. This discrepancy may be explained by the complex relationship between these factors. For instance, a recent study provided evidence of compensatory mitochondrial responses to lipid‐induced insulin resistance, indicating that the relationship between mitochondria and insulin resistance is multifaceted.^9^

In a non‐psychiatric population, it was shown that lower mtDNA CN correlates with global cognitive decline^14^ and a higher incidence of neurodegenerative disease.^16^ The underlying mechanisms of this association possibly involve oxidative stress, altered calcium homeostasis, and neuronal apoptosis.^8^ The interplay of mitochondrial dysfunction with proteins like amyloid‐beta and tau could also contribute to neurodegeneration.^18^ Our earlier research suggested that BD patients on valproate with higher mtDNA CN exhibited superior executive function, similar to our results in the current study.^19^

Addressing the problem of insulin resistance is increasingly recognized as a vital part of treating mood disorders, as evidence consistently points to improved outcomes.^20^, ^21^ Our study further supports this idea, as our data imply that insulin resistance and mitochondrial dysfunction could be potentially useful therapeutic targets to mitigate cognitive deterioration in BD. As such, interventions like insulin‐sensitizing medications, lifestyle adjustments, and nutritional supplementation could potentially be useful to enhance mitochondrial function and cognitive health. Medication review and cognitive therapies aimed at metabolic wellness may also enhance cognitive outcomes for BD patients.

Our mediation analysis revealed a significant indirect effect of mtDNA CN on the relationship between insulin resistance and executive dysfunction. However, the absence of a direct effect of insulin resistance on cognitive outcomes (indicated by non‐significant ADE) raises intriguing questions. For instance, a possible explanation is that while mtDNA CN mediates the relationship between insulin resistance and executive function, other factors may also influence the relationship. To address this idea in future studies, it will be important to evaluate potential confounders or moderators that could clarify the lack of a direct effect. For example, variables such as age, illness duration, medication type and dosage, lifestyle factors (e.g., diet and physical activity), and inflammatory markers may play roles in modifying or confounding the relationship between insulin resistance and cognitive performance. Exploring the influence of these factors may uncover additional pathways that affect both insulin resistance and cognitive function independent of mtDNA CN. Additionally, it would be helpful to examine whether specific subgroups, such as patients with different BD subtypes or distinct metabolic profiles, might exhibit different associations between metabolic health, mitochondrial function, and cognitive outcomes. Understanding such subgroup‐specific variations could reveal whether the lack of a direct effect observed in our study reflects unique characteristics of certain patient populations or a more generalized pattern across the BD population.

Our study has several limitations. First, the sample size was relatively small. Therefore, a cautious interpretation should be made of our results, including the identified mediation effects. Future studies should be conducted with a larger sample size to enhance statistical power and generalizability. Second, the study was cross‐sectional in nature, so causality could not be inferred. A longitudinal design in future research could better establish causal relationships by tracking changes over time. Third, the sensitivity analysis suggested our results may be impacted by unmeasured confounders, such as illness duration, blood cell counts, or inflammatory markers. Controlling for these additional covariates would help clarify the direct and indirect effects of insulin resistance on cognition. Fourth, we used mtDNA CN measured in peripheral leukocytes as a proxy for brain tissue mtDNA CN; however, differences may exist in mitochondrial function and mtDNA content across tissue types.^14^, ^16^ Future studies could address this issue by using brain‐specific mtDNA CN measurements if feasible, providing a more accurate assessment of mitochondrial status in the central nervous system. Additionally, the value of the leukocyte mtDNA CN proxy could be better understood by conducting intervention studies that focus on reducing insulin resistance through lifestyle changes or insulin‐sensitizing medications, while also tracking mtDNA CN and cognitive outcomes. Most importantly, such studies would help to determine if improving metabolic and mitochondrial health directly benefits cognitive function in patients with BD.

In conclusion, our data showed that insulin resistance may lower mtDNA CN in blood, which may in turn have detrimental indirect effects on executive function in euthymic BD patients. Further studies are warranted to track how changes in mtDNA CN correlate with both metabolic markers like insulin resistance and cognitive performance over time. More work is also needed to establish whether interventions that target metabolic and mitochondrial health can concretely improve cognitive outcomes in patients with BD.

## CONFLICT OF INTEREST STATEMENT

All authors declare no conflict of interest. The funding institutions of this study had no further role in study design, data collection, analysis, preparation of this manuscript, or the decision to submit for publication. The authors report no financial relationships with commercial interests.

## Data Availability

The data used to support the findings of this study are available from the corresponding author upon request.
